# Impact of *Nigrospora oryzae*‐Derived Natural Products on Photosynthesis and Oxidative Stress in *Eichhornia crassipes*

**DOI:** 10.1111/ppl.70104

**Published:** 2025-02-03

**Authors:** Chamroon Laosinwattana, Nutcha Manichart, Pattharin Wichittrakarn, Kaori Yoneyama, Montinee Teerarak, Hataichanok Passara

**Affiliations:** ^1^ School of Agricultural Technology, King Mongkut's Institute of Technology Ladkrabang Bangkok Thailand; ^2^ International Academy of Aviation Industry, King Mongkut's Institute of Technology Ladkrabang Bangkok Thailand; ^3^ Department of Biochemistry and Molecular Biology Saitama University Saitama Japan

## Abstract

Interest in natural herbicides has been growing due to government policies restricting synthetic herbicide use in many countries. In that regard, this study investigates the potential of *Nigrospora oryzae* extract as a natural herbicide against the aquatic invasive weed *Eichhornia crassipes*. A stable formulation was developed with a droplet size of 36.44 ± 0.36 nm and a zeta potential of ‐62.59 mV. Pot‐based experiments revealed the *N. oryzae* extract induced 38.33% phytotoxicity within 24 hours, increasing to 84.72% by 28 days post‐treatment. Scanning electron microscopy demonstrated morphoanatomical changes in epidermal tissue and stroma of *E. crassipes*, such as erosion of epicuticular waxes and degeneration of epidermis cells. The treatment significantly reduced the photosynthetic pigment content while increasing hydrogen peroxide (46.26%), malondialdehyde (17.49%), and proline (19.16%) levels, causing cellular electrolyte leakage. Activities of superoxide dismutase, catalase, ascorbate peroxidase, and guaiacol peroxidase were significantly elevated (*p*<0.05), indicating oxidative damage. These findings demonstrate that *N. oryzae* extract can disrupt growth and photosynthesis and induce oxidative stress in *E. crassipes*, suggesting its potential as a source of natural herbicide for industrial application.

## 1 INTRODUCTION

*Eichhornia crassipes* (Mart.) Solms, commonly known as water hyacinth, is one of the most problematic invasive aquatic plants worldwide. Traditional management strategies for water hyacinth control have primarily relied on mechanical removal and chemical synthetic herbicides, with glyphosate‐based, bipyridyl‐based (paraquat), and chlorophenoxy herbicides being widely used (Waltham and Fixler, 2017; Almeida et al., 2016). However, increasing concerns about environmental and health impacts have led some countries to ban or restrict the use of certain herbicides. For instance, Thailand has implemented restrictions on glyphosate and paraquat, resulting in a shift towards greater use of chlorophenoxy herbicides, particularly 2,4‐dichlorophenoxyacetic acid (2,4‐D), 2,4,5‐trichlorophenoxyacetic acid (2,4,5‐T), and 2‐methyl‐4‐chlorophenoxyacetic acid (MCPA) (Kurniadie et al., 2023; Chomchalow, 2011). These chemical control methods can be costly, labor‐intensive, and potentially harmful to non‐target organisms and the lentic ecosystem.

Recently, a new approach has emerged for developing sustainable and environmentally friendly methods to manage aquatic weeds. Natural products derived from microorganisms, particularly fungi, have gained attention as potential sources of novel natural herbicides (Namasivayam Pandian et al., 2023; Babu et al., 2003). Among these, extracts from the fungal genus *Nigrospora* have shown promising results; members of this genus produce metabolites with various biological activities, including plant growth inhibition and herbicidal properties (Salvatore et al., 2024). Recent studies have demonstrated the phytotoxic effects of *N. oryzae* metabolites on *Rumex dentatus* and *Sonchus oleraceus* (Mohammed and Badawy, 2020). Crude extracts from *N. oryzae* cultures have likewise demonstrated significant phytotoxic activity in multiple studies. Tanaka et al. (1997) found that nigrosporins A and B in methanol extracts effectively suppressed the growth of *Echinochloa crus‐galli*, a problematic weed in rice cultivation, as well as *Setaria viridis* and *Abutilon theophrast*. Naseem et al. (2016) reported that carbon tetrachloride and chloroform extracts significantly inhibited root and shoot growth in *Parthenium hysterophorus*. Salvatore et al. (2024) reported the isolation and structural elucidation of 3‐(hydroxymethyl)‐2‐methylpentane‐1,4‐diol (nigrosphaeritriol) and 3‐(1‐hydroxyethyl)‐4‐methyltetrahydrofuran‐2‐ol (nigrosphaerilactol) from the phytotoxic extract used to control buffelgrass. Azaphilone phytotoxin derivatives, such as nigbeauvins and nigcollins, were also identified in the metabolites of *N. oryzae* cultures (Wu et al., 2022). These azaphilone fungi‐derived polyketide secondary metabolites have shown potent phytotoxicity in numerous studies (Wang et al., 2022; Xu et al., 2021). Several phytochemicals were detected in both the broth culture and ethyl acetate mycelial extracts of *Nigrospora* sp., which tested positive for glycosides, alkaloids, flavonoids, terpenoids, sterols, indoles, and phenols (Notarte et al., 2019). Later findings have further suggested the presence of both extracellular and intracellular phytotoxic metabolites in *Nigrospora* (Xu et al., 2021).

Crude extracts often exhibit multiple modes of action due to the diverse array of allelochemicals they contain (Berestetskiy, 2023; Laosinwattana et al., 2007). Many herbicides induce oxidative stress in target plants, triggering a cascade of physiological and biochemical changes that ultimately lead to plant death (Sule et al., 2022). The generation of reactive oxygen species (ROS) is often a primary mechanism by which these compounds exert their phytotoxic effects (Traxler et al., 2023). A diverse composition also enables extracts to have a multi‐targeting effect and synergistic interactions, conferring efficacy against various targets (Bordin et al., 2021; Cimmino et al., 2015). However, crude‐extract‐based herbicides may also face some challenges in development, such as poor stability, solvent toxicity, and low solubility (Berestetskiy, 2023). These issues may be resolved through the use of formulation agents, which can significantly affect a product's physicochemical properties and enhance its stability and overall efficacy (Mesnage, 2021). Especially, smaller particle size and lower polydispersity index (PI) improve wettability, stability, and bioavailability compared to conventional suspension concentrates. This study aims to characterize the physicochemical properties of natural products derived from *N. oryzae* extract and their allelopathic herbicidal effects on *E. crassipes*. It primarily focuses on formulation optimization, efficacy assessment, and mode of action as indicated by oxidative stress indicators, cellular responses, and effects on photosynthetic pigment contents in treated plants. All told, this research presents the potential of *N. oryzae* extract as an eco‐friendly natural herbicide for water hyacinth management. Its findings contribute to the development of sustainable aquatic weed management strategies and advance our knowledge of fungal‐based natural product herbicides.

## 2 MATERIALS AND METHODS

### 2.1 Fungi, molecular identification, and phylogenetic analyses

Fungi were previously isolated from diseased *Echinochloa crus‐galli* (L.) T. Beauv. weeds collected in September 2021 from the Ladkrabang district of Bangkok, Thailand (geographic coordinates: 13.726725, 100.780125). Fungal genomic DNA was isolated using the CTAB method. The internal transcribed spacer (ITS) region of fungal ribosomal DNA (rDNA), translation *ELONGATION FACTOR 1‐ALPHA* (*TEF‐*1), and *β‐TUBULIN* (*TUB2*) were amplified by polymerase chain reaction (PCR) using primers from the protocol previously established by O'Donnell et al. (2010) and Carbone and Kohn (1999). Sequencing of PCR products was carried out by Marcogen Inc., Seoul, South Korea. To identify cultured fungi, the ITS, *TEF‐1*, and *TUB2* sequences were combined and similarity searches performed using BLAST against the available sequences at NCBI (https://blast.ncbi.nlm.nih.gov). Multiple sequence alignment was performed with MUSCLE 14 and improved where necessary using BioEdit v. 7.2. Phylogenetic analysis of the combined ITS, *TEF‐1*, and *TUB2* data was performed in MEGA 11 using the maximum likelihood (ML) method with 1,000 bootstrap replications. All sequence data generated in this study were deposited in NCBI GenBank.

### 2.2 Fungal extract and preparation of the *N. oryzae*‐based natural product

The *N. oryzae* isolates were cultured and collected following Manichart et al. (2023). Briefly, dried mycelia were ground and extracted (ratio 1:10) with 75% v/v ethanol (EtOH) for 24 hours before ultrasonic oscillation for 30 minutes at 50°C. The supernatants were then filtered through cotton and re‐filtered using Whatman No. 1 filter paper (Whatman Inc.). To obtain the crude extract, the resultant filtrate solutions were evaporated using a rotary vacuum evaporator (BUCHI Rotavapor R255, BUCHI) at 45°C under partial vacuum. The *N. oryzae*‐based natural product was formulated at a 3:4:3 ratio (crude extract: dimethylformamide [DMF]:Tween 80; w/w). Briefly, the sticky fungal crude extract and DMF were mixed with a magnetic stirrer at 1,500 rpm for 20 minutes. Tween 80 was then added to the mixture and stirred continuously for three minutes. Water was added, and the solution was stirred constantly for an additional ten minutes. After homogenization, this stock solution was kept at 4°C, and its physicochemical parameters were assessed in the following experiments.

### 2.3 Characterization of the formulant

The *N. oryzae*‐based natural product was evaluated in terms of particle size and polydispersity index (PI) by dynamic light scattering (DLS) using a Nanoplus 3 (Micromeritics). Measurement conditions consisted of a fixed scattered angle of 165° and temperature of 25°C. The zeta (ζ) potential, reflective of electrophoretic properties, was assayed to predict formulation stability due to electrostatic repulsion (Campolo et al., 2020). Measurement conditions for ζ‐potential included a fixed scattered angle of 15° and temperature of 25°C. Five replicates of each measurement were collected, and calculations were performed using the program Nanoplus version 5.10/3.00.

### 2.4 Herbicidal activity assay and symptom evaluation in greenhouse conditions

*E. crassipes* was collected from the Ladkrabang district of Bangkok, Thailand. This study complied with relevant institutional, national, and international guidelines and legislation. Following collection, the aquatic macrophytes were carefully arranged and kept in water‐filled containers placed in buckets under greenhouse conditions (average temperature 28‐30°C, natural light, relative humidity 64‐69%). The *N. oryzae*‐based natural product (NO product) was applied to *E. crassipes* leaves manually with the wrapping technique, in which leaf surfaces (adaxial and abaxial) and petioles were coated with extract preparations (2.0, 4.0, and 8.0% w/v active ingredient). Water and a surfactant mixture were used as control agents. Regarding the experiment design, a completely random design was employed with ten replicates. After treatment, the phytotoxic effects were evaluated according to the scale adopted by the European Weed Research Council (Meseldžija et al., 2020). The treated plants were continuously monitored, and visual toxicity symptoms were graded on a scale of 0% to 100%, where ‘0’ means a lack of toxicity and ‘100’ resulted in plant death. For micromorphological (scanning electron microscopy; SEM) analysis, leaf samples were first dehydrated in an ethanolic series that extended up to 100%, then placed on pin stubs with carbon tape and sputter coated with gold (Balzers, SCD 040). Observations and photographs were obtained using a scanning electron microscope (JEOL InTouchScope™, JSM‐IT500HR, JED‐2300) at the Scientific and Technological Research Equipment Centre (STREC) at Chulalongkorn University, Thailand. Later, the leaf surfaces were imaged using the SMILE VIEW™ Lab integrated data management software.

### 2.5 Identification of *N. oryzae* extract constituents by gas chromatography/mass spectrometry

Constituents of the *N. oryzae* extract were identified using gas chromatography/mass spectrometry. A Scion 436 gas chromatograph was coupled to a triple quad (Bruker) mass spectrometer. Operating parameters were as follows: helium flow rate of 1 ml/minute; detection range of 30–500 amu; starting oven temperature of 50°C (2 minutes); ramping to 250°C (20°C minute^−1^); and then holding for 18 minutes. The HP‐5MS capillary column (30 m, film 0.25 μm, ID 0.25 mm) was filled with 1 mL sample in splitless mode. The temperature of the transfer line was 250°C and that of the ion source 230°C. Individual ingredients were identified by comparison of obtained mass spectra (molecular mass and fragmentation pattern) with the internal reference library (National Institute of Standards and Technology, NIST, 2014). Components were quantified in terms of the percentage peak area relative to total peak area.

### 2.6 Mechanism of action

#### 2.6.1 Photosynthetic pigments determination

To extract photosynthetic pigments, treated leaves (0.5 g FW) were mashed in 80% v/v acetone for three hours under dark conditions at room temperature. Then, chlorophyll *a*, *b*, and carotenoid contents were determined by the spectrophotometric method at 470, 647, and 663 nm using a UV/vis spectrophotometer (Thermo Fisher Scientific), and the pigment contents were calculated using the Lichtenthaler (1987) equation.

$$\text{Chlorophyll}\;a=12.25\times A_{663}-2.79\times A_{647}$$

$$\text{Chlorophyll}\;b=21.50\times A_{647}-5.10\times A_{663}$$

$$\text{Carotenoids}=\left(1,000\times A_{470}–1.82\times \mathrm{Chl}\;a–85.02\times \mathrm{Chl}\;b\right)/198$$

#### 2.6.2 Relative electrolyte leakage

Electrolyte leakage was assessed using the method of Singh et al. (2007). Five leaf discs from each treated plant were floated on 5.0 mL of deionized water after either 30 minutes at 40°C (EC_1_) or boiling at 100°C for 15 minutes (EC_2_), and the conductivity was measured using a Consort C3010 multi‐parameter analyzer (Consort). Relative electrolyte leakage (REL) was calculated by the formula: REL (%) = (EC_1_/EC_2_)×100

#### 2.6.3 Determination of oxidative stress markers

Oxidative damage in *E. crassipes* was assessed by measuring malondialdehyde (MDA), hydrogen peroxide (H_2_O_2_), and proline levels. Leaf samples were collected from the above‐water parts of ten plants. Approximately 0.5 g of leaf material was ground in 4 mL of 0.1% ice‐cold trichloroacetic acid, then centrifuged at 9600 ×*g* (4°C), with the supernatant used for stress marker determination. Lipid peroxidation was determined by estimating MDA concentration using the thiobarbituric acid reactive substances assay, according to Kramer et al. (1991). Absorbance was measured at 532 nm and 600 nm, and the MDA concentration was calculated using an extinction coefficient of 155 m M^−1^ cm^−1^. H_2_O_2_ levels were determined according to Alexieva et al. (2001) based on a standard curve prepared with known concentrations of H_2_O_2_. Free proline was determined by the acid ninhydrin assay (Bates et al., 1973), with proline content determined from a standard curve.

#### 2.6.4 Enzyme extraction and assays

Crude enzymatic extracts were prepared following the method described by Mir et al. (2018). Briefly, 6.0 mL of 50 mM phosphate buffer containing polyvinylpolypyrrolidone and 0.5 mM ethylenediaminetetraacetic acid was used to extract treated *E. crassipes* leaf samples (1.0 g FW). The homogenates were centrifuged, and the resulting supernatants were used for total soluble protein estimation, which is essential for calculating enzyme‐specific activity and conducting antioxidant enzyme assays, including superoxide dismutase (SOD; EC 1.15.1.1), catalase (CAT; EC 1.11.1.6), and guaiacol peroxidase (GPX; EC 1.11.1.7). Protein concentration in the enzyme extracts was determined colorimetrically using the Bradford method (Bradford, 1976), with bovine serum albumin as the standard, by measuring absorbance at 595 nm. SOD was measured spectrophotometrically at 560 nm based on the inhibition of nitro‐blue tetrazolium (NBT) photoreduction (Kamran et al., 2021). SOD activity was expressed as units per mg protein, where one unit is defined as the amount of enzyme inhibiting 50% of NBT photoreduction. CAT activity was determined by monitoring the decomposition of H_2_O_2_ at 240 nm according to Chen and Zhang (2016). CAT activity was expressed as units per mg protein, with one unit defined as the amount of enzyme that decreases the absorbance at 240 nm by 0.1 per minute. GPX activity was measured using guaiacol as a substrate according to He et al. (2014). The increase in absorbance at 470 nm due to guaiacol oxidation was recorded every 15 seconds for 1 minute. GPX activity was expressed as units per mg protein, with one unit defined as the amount of enzyme that increases the absorbance at 470 nm by 0.01 per minute. For ascorbate peroxidase (APX; EC 1.11.1.11) activity, the extraction was adapted from the method described by Kumar (2022) with slight modifications. Briefly, 1 g of fresh, cleaned tissue samples were homogenized in 10 mL of freshly prepared 50 mM phosphate buffer (pH 7.0) containing 0.5 mM L‐ascorbic acid to stabilize APX during extraction. The homogenates were centrifuged, and the resulting supernatants were collected as crude enzyme extracts. APX activity was determined by monitoring the decrease in absorbance at 290 nm over 2 minutes, attributed to the H_2_O_2_−dependent oxidation of ascorbate. The activity was expressed as μmol min^−1^ mg^−1^ protein.

### 2.7 Statistical analysis

For each sample, four replicates were collected for biochemical analyses and ten replicates for growth traits. The results are shown as means. All data were analyzed using SAS and subjected to analysis of variance (ANOVA) and comparison of means by Tukey's multiple range test (*p* ≤ 0.05). Means followed by the same letter(s) are not significantly different.

## 3 RESULTS AND DISCUSSION

### 3.1 Fungal taxonomy

**FIGURE 1:**
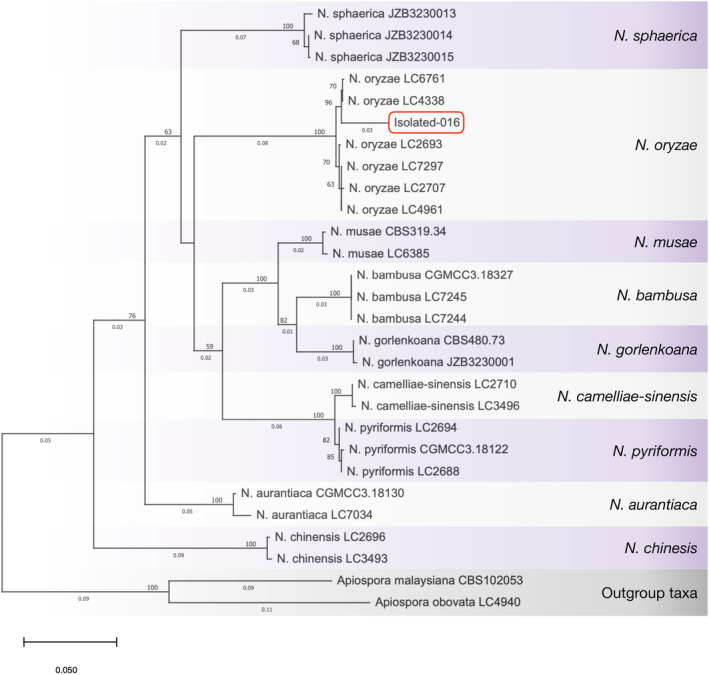
Multiple‐gene maximum likelihood phylogenetic tree based on the combined ITS, *TEF‐1*, and *TUB2* sequence alignment. The red circle highlights the sequence obtained in this study. The tree is drawn to scale, with branch lengths corresponding to the number of substitutions per site (given below the branches). Bootstrap support values for ML (≥ 50%) are given at the nodes.

PCR amplification and sequencing of the ITS, *TEF‐1*, and *TUB2* regions revealed nucleotide sequences of 673, 493, and 394 bp, respectively. NCBI‐BLASTn analysis showed the highest pairwise similarity (98.30%) with *Nigrospora oryzae* isolate 2694, accession number EU272498. Table 1 lists the NCBI accession numbers for the sequence from this study and published sequences used for phylogenetic analysis. Phylogenetic analysis based on the combined ITS, *TEF‐1*, and *TUB2* sequence data revealed the fungal isolate‐016 obtained from diseased *Echinochloa crus‐galli* in Thailand to cluster within the *N. oryzae* clade (Figure 1). The ML tree showed strong bootstrap support (≥ 50%) for the placement of isolate‐016 within this clade, which includes other *N. oryzae* strains isolated from various plant hosts such as *Oryza sativa*, *Nelumbo* sp., and *Camellia* sp. (Liu et al., 2021; Hao et al., 2020). Based on these two methods, we conclude that isolate‐016 belongs to *N. oryzae*.

**TABLE 1:** *Nigrospora* strains and details of sequences used in the molecular phylogenetic analysis of this study.

Taxa	Strain/Isolate	Host	GenBank accession numbers		
			*ITS*	*TEF‐1*	*TUB2*
**Outgroup**					
*Arthrinium malaysianum*	CBS 102053	–	NR120273	KF145030	KF144988
*Arthrinium obovatum*	LC 4940	–	KY494696	KY705095	KY705166
***Nigrospora* species**					
*N. sphaerica*	JZB 3230013	*Cirsium setosum*	MN495951	MN544642	MN549390
*N. sphaerica*	JZB 3230014	*Phragmites australis*	MN495952	MN544643	MN549391
*N. sphaerica*	JZB 3230015	*Fraxinus* sp.	MN495953	MN544644	MN549392
*N. pyriformis*	CGMCC 3.18122	*Citrus sinensis*	KX985940	KY019290	KY019457
*N. pyriformis*	LC 2688	*Lindera aggregate*	KX985941	KY019297	KY019468
*N. pyriformis*	LC 2694	*Rubus reflexus*	KX985945	KY019300	KY019472
*N. oryzae*	LC 6761	*Oryza sativa*	KX986056	KY019376	KY019574
*N. oryzae*	LC 7297	*Nelumbo* sp. (leaf)	KX985936	KY019400	KY019605
*N. oryzae*	LC 2693	*Neolitsea* sp.	KX985944	KY019299	KY019471
*N. oryzae*	LC 2707	*Rhododendron simiarum*	KX985954	KY019307	KY019481
*N. oryzae*	LC 4338	*Camellia* sp.	KX986008	KY019349	KY019532
*N. oryzae*	LC 4961	*Pittosporum illicioides*	KX986031	KY019358	KY019553
***N. oryzae***	**016**	***Echinochloa crus‐galli***	**PP532837**	**PP565361**	**PP565362**
*N. musae*	CBS 319.34	*Musa paradisiaca* (fruit)	KX986076	KY019419	KY019455
*N. musae*	LC 6385	*Camellia sinensis*	KX986042	KY019371	KY019567
*N. gorlenkoana*	CBS 480.73	*Vitis vinifera*	KX986048	KY019420	KY019456
*N. gorlenkoana*	JZB 3230001	*Cirsium setosum*	MN495939	MN544645	MN549381
*N. chinensis*	LC 2696	*Lindera aggregata*	KX985947	KY019424	KY019474
*N. chinensis*	LC 3493	*Camellia sinensis*	KX985984	KY019434	KY019509
*N. camelliae‐sinensis*	LC 2710	*Castanopsis* sp.	KX985957	KY019310	KY019484
*N. camelliae‐sinensis*	LC 3496	*Camellia sinensis*	KX985985	KY019327	KY019510
*N. bambusae*	CGMCC 3.18327	Bamboo (leaf)	KY385307	KY385313	KY385319
*N. bambusae*	LC 7244	Bamboo (leaf)	KY385306	KY385314	KY385320
*N. bambusae*	LC 7245	Bamboo (leaf)	KY385305	KY385315	KY385321
*N. aurantiaca*	CGMCC 3.18130	*Nelumbo* sp. (leaf)	KX986064	KY019295	KY019465
*N. aurantiaca*	LC 7034	*Musa paradisiaca*	KX986093	KY019394	KY019598

The sequence generated in this study is in bold typeface.

### 3.2 Characterization of the *N. oryzae*‐based natural product

**FIGURE 2:**
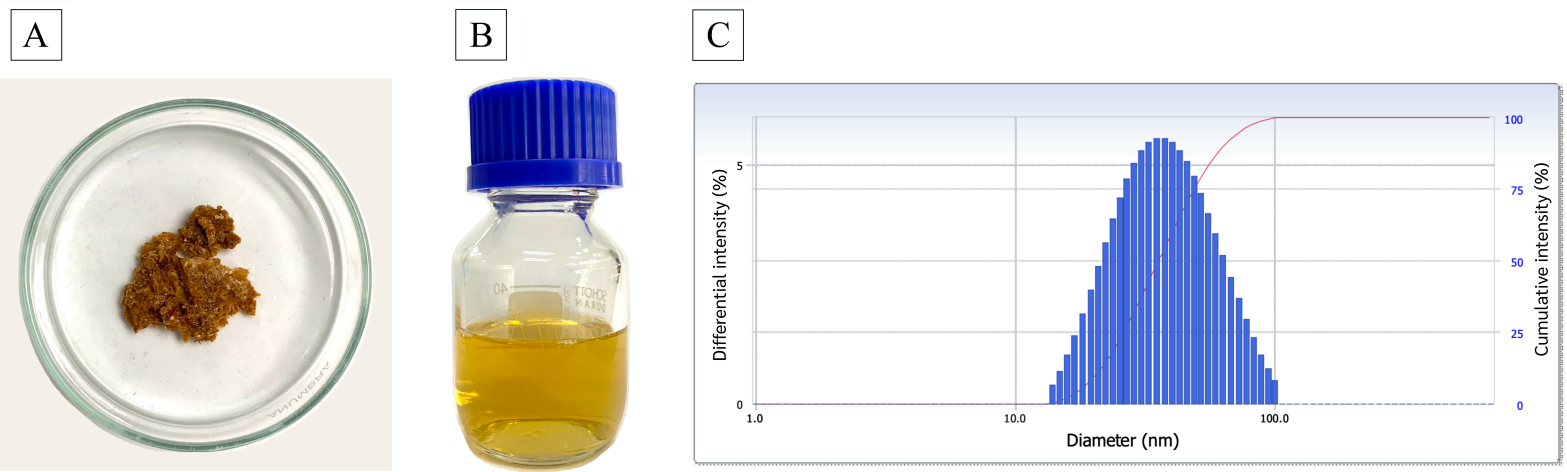
Visual appearance of the crude extract (A) and the *N. oryzae*‐based natural product (B), along with its particle size distribution (C) according to dynamic light scattering (DLS).

The formulation of natural products is important to their stability, bioavailability, and overall efficacy as natural herbicides. Specifically, the use of formulation agents such as DMF and Tween 80 can significantly affect the physicochemical properties of bioactive compounds by influencing key parameters such as particle size, PI, and ζ‐potential (López‐Cabeza et al., 2022). Here, natural product formulations were prepared from *N. oryzae* crude extract (Figure 2A) and a surfactant mixture using a low‐energy technique. The resulting formulations appeared translucent with a yellow‐orange tint, showing no phase separation, flocculation, or coalescence (Figure 2B). Characterization of the *N. oryzae*‐based natural product by DLS yielded the droplet size distribution shown in Figure 2C, with a particle size of 36.44 ± 0.36 nm and a PI of 0.247 ± 0.006. The ζ‐potential, determined by electrophoresis, was approximately ‐62.59 mV. Particle size is a factor in determining the bioavailability and activity of natural products; nano‐formulations with smaller particle sizes generally exhibit enhanced solubility and improved penetration into plant tissues, potentially leading to greater herbicidal efficacy (Sun et al., 2022). Meanwhile, PI indicates the homogeneity of a formulation. The obtained PI values were less than 0.5, indicating a homogeneous droplet size distribution suitable for agricultural applications (Mustafa and Hussein, 2020). Finally, a ζ‐potential with an absolute value greater than 30 mV indicates a stable formulation. Higher absolute ζ‐potential values correlate with increased formulation stability and better adhesion to plant surfaces (Afzal et al., 2021).

### 3.3 Natural product effect on *E. crassipes* control

**FIGURE 3:**
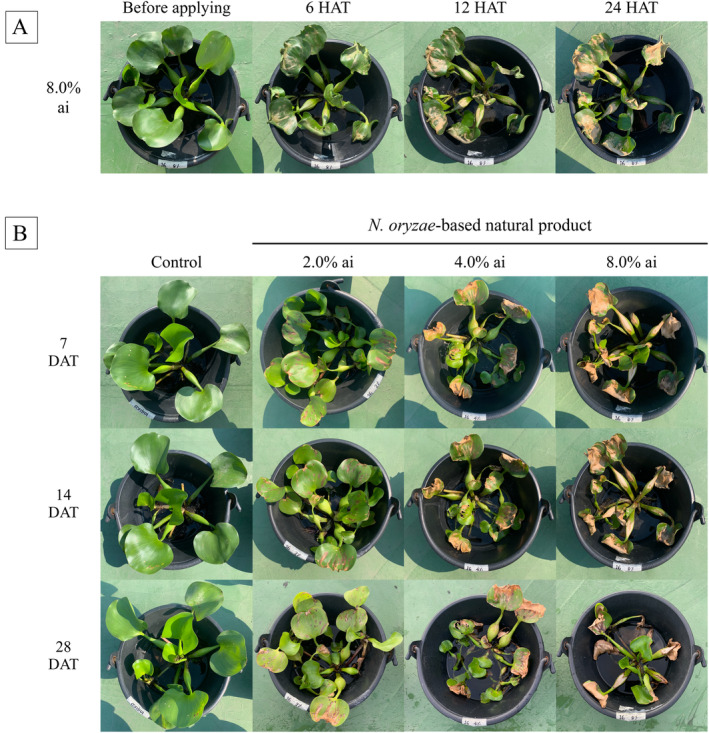
Phytotoxicity symptoms observed in *E. crassipes* plants exposed to the *N. oryzae*‐based natural product. (A) Symptoms observed 24 HAT with 8% of the active ingredient. (B) Symptoms observed at 7, 14, and 28 DAT. HAT, hours after treatment; DAT, days after treatment; ai, active ingredient.

Application of the *N. oryzae*‐based natural product to *E. crassipes* in the greenhouse experiment revealed significant phytotoxic effects, demonstrating the potential of this fungal extract as a natural herbicide. Specifically, dose‐dependent visual toxicity symptoms were observed at 1, 7, 14, and 28 days after treatment (DAT). At 8.0% active ingredient, the extract induced 38.33% toxicity by 24 hours after treatment (HAT) (Figure 3A), increasing remarkably to 47.22% within 7 DAT (Figure 3B). This rapid induction of phytotoxicity symptoms suggests that the allelochemicals in the extract are quickly absorbed and translocated within plant tissues. Similar rapid phytotoxicity has been reported for other fungal‐derived allelochemicals, such as altheichin from *Alternaria eichorniae*, which showed significant effects on water hyacinth within 12 hours of application (Robeson et al., 1984). The visual symptoms observed here, including leaf burning, wilting, and necrosis, are consistent with the effects of other fungal‐derived herbicidal compounds on *E. crassipes* (Namasivayam Pandian et al., 2023). Additionally, growth inhibition was evident from the significant reduction (*p*<0.05) in dry weights of treated plants (Table 2). The 8.0% active ingredient treatment resulted in the lowest dry weights (35.0% less than control). This aligns with findings from Yirefu et al. (2017), who reported similar growth inhibition in water hyacinth when treated with extracts from *A. tenuissima*, *Fusarium oxysporum*, *F. equiseti*, and *Botryosphaeria* sp. Application of these extracts caused reduction in weed fresh weight, dry weight, plant height, and root length, ranging between 11‐67%, 22‐72%, 15‐55%, and 12‐50%, respectively.

**TABLE 2:** Phytotoxicity of the *N. oryzae*‐based natural product on *E. crassipes* weeds under greenhouse conditions.

Treatment	Visible phytotoxicity level (% over control)				DW/ plant (g)
	1 DAT	7 DAT	14 DAT	28 DAT	
Control	–	–	–	–	69.50 b
Surfactant mixture	0.00 a	4.17 b	6.94 a	5.56 a	75.00 b
*N. oryzae* extract					
2.0%	2.78 a	9.72 a	27.22 b	38.61 b	66.50 b
4.0%	10.28 b	26.39 c	49.44 c	63.06 c	46.00 a
8.0%	38.33 c	47.22 d	72.22 d	84.72 d	35.00 a
C.V. (%)	14.01	6.69	7.54	8.91	24.83

Mean values (n = 10) with the same letter(s) within a column are not significantly different according to Tukey's multiple range test (*p*<0.05).

### 3.4 Microanatomy change of *E. crassipes* weed leaves

**FIGURE 4:**
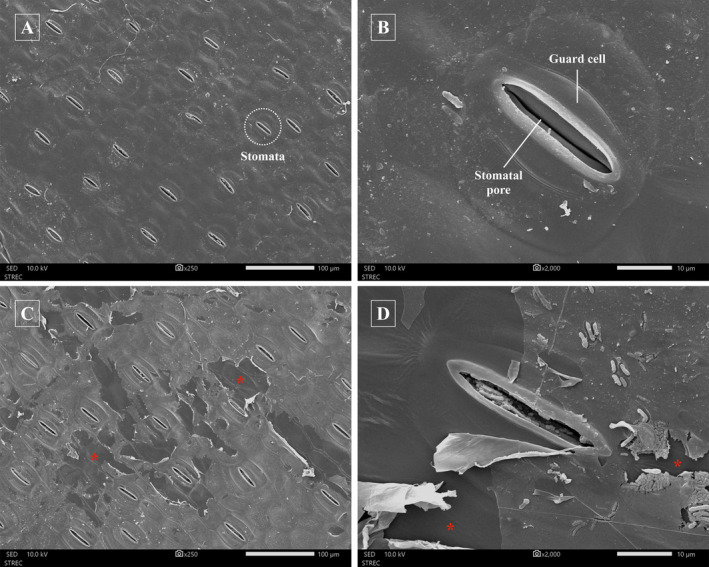
Scanning electron microscopy of the adaxial surface of *E. crassipes* leaf blades 24 hours after *N. oryzae*‐based natural product application. (A‐B) Control plants showing epidermis cells, guard cells, and stomata; (C‐D) Treated plants, 24 hours after exposure to the *N. oryzae*‐based natural product, displaying erosion of epicuticular wax (red stars).

SEM analysis revealed morphological changes of *E. crassipes* leaf blades 24 hours after application of the *N. oryzae*‐based natural product. The leaf surface, with its cuticle and epicuticular waxes (Figure 4A‐B), is the primary site of herbicide absorption and a key determinant of herbicide tolerance (Carr et al., 1986; Chotsaeng et al., 2018), with epidermal cells reportedly being the main facilitators of herbicide uptake (Kerstiens, 1996; Carr et al., 1986). Treated leaves showed significant degradation and erosion of epicuticular waxes (Figure 4C‐D). A lack of epicuticular wax was also reported in epidermal cells of *Triticum aestivum* treated with the herbicide propoxycarbazone‐sodium (Yilmaz and Dane, 2012). Such damage leads to separation of the waxes from the underlying epidermis, compromising the protective layer. The resulting thinner, disrupted leaf surface likely facilitates even more herbicide penetration and absorption. Moreover, epicuticular wax plays a crucial role in retaining water within the plant (Muhammad et al., 2020), and their disruption may cause water loss and wilting, exacerbating the herbicide's effects. The detrimental effects on leaf morphology due to herbicide action observed here, such as erosion of epicuticular waxes, cuticle rupture, and degeneration of epidermal and parenchymal cells, were also reported in other studies on glyphosate (Santos et al., 2019), dichlorprop (Qiu et al., 2022), clomazone, and saflufenacil (David et al., 2020).

### 3.5 Identification of constituents from *N. oryzae* crude extract

GC‐MS analysis of the *N. oryzae* mycelial extract detected 42 compounds, comprising 91.58% of the total crude aqueous ethanolic extract (Table 3). The spread of detected compounds is consistent with the prior report of Prasher and Dhanda (2017), who found organosilicon derivatives in *N. sphaerica* extracts. In this *N. oryzae* mycelial extract, the major constituents are sugar alcohols and derivatives, identified as hexitol (33.08%), a polyol important to microorganisms for osmo‐protection and carbohydrate storage (Ravikumar et al., 2022) whose abundance is influenced by growth conditions (Galdiero et al., 2021). This is similar to *N. sphaerica* mycelium extract, which also contains arabitol (17.01%) (Salvatore et al., 2024).

**TABLE 3:** Constituents of the *N. oryzae* crude aqueous ethanolic extract.

No.	Chemical constituent	M.F.^a^	R.T.^b^	%^c^
1	Hexamethylcyclotrisiloxane	C_6_H_18_O_3_Si_3_	3.737	0.310
2	2,4,6‐cycloheptatrien‐1‐one, 3,5‐bis‐trimethylsilyl‐	C_13_H_22_OSi_2_	3.886	0.113
3	N,N,6‐trimethyl‐1,3,2‐oxathiaphosphinan‐2‐amine 2‐sulfide	C_6_H_14_NOPS_2_	4.910	0.103
4	1‐dimethyl(butyl)silyloxybutane	C_10_O_24_OSi	5.381	0.269
5	Butoxyacetic acid, TMS derivative	C_9_H_20_O_3_Si	5.451	0.951
6	4‐methylmannitol	C_7_H_16_O_6_	5.471	0.992
7	2,5‐dihydroxyacetophenone, 2TMS derivative	C_14_H_24_O_3_Si_2_	5.565	0.110
8	Putrescine	C_4_H_12_N_2_	5.712	0.136
9	Hexanoic acid, ethyl ester	C_8_H_16_O_2_	5.903	0.575
10	Malic acid	C_4_H_6_O_5_	6.049	1.941
11	Pyrrolidin‐2‐one	C_4_H_7_NO	6.358	0.106
12	L‐lysine	C_6_H_14_N_2_O_2_	6.383	0.271
13	2(3H)‐furanone, dihydro‐4‐hydroxy‐	C_4_H_6_O_3_	7.015	0.183
14	1‐methoxy‐1‐methylsilinane	C_7_H_16_OSi	7.108	0.478
15	Dianhydromannitol	C_6_H_10_O_4_	7.470	0.332
16	Conhydrin	C_8_H_17_NO	9.034	1.740
17	1,5‐diazocine, octahydro‐1,5‐dinitroso‐	C_6_H_12_N_4_O_2_	9.117	0.207
18	Allyl(2‐tetrahydrofurylmethoxy)dimethylsilane	C_10_H_20_O_2_Si	9.193	1.665
19	4‐methyl(trimethylene)silyloxyoctane	C_12_H_26_OSi	9.373	0.516
20	Desulphosinigrin	C_10_H_17_NO_6_S	9.838	0.440
21	2‐hexadecanol	C_16_H_34_O	9.904	0.214
22	1‐butyl(dimethyl)silyloxypropane	C_9_H_22_OSi	10.019	1.257
23	Heptose	C_7_H_14_O_7_	10.465	1.253
24	D‐gala‐l‐ido‐octonic amide	C_8_H_17_NO_8_	10.590	0.284
25	2,3‐diacetyloxypropyl dodecanoate	C_19_H_34_O_6_	10.623	0.222
26	Cyclopropanetetradecanoic acid, 2‐octyl‐, methyl ester	C_26_H_50_O_2_	10.724	1.110
27	9‐hexadecenol	C_16_H_32_O	10.84	1.860
28	Lithic acid	C_5_H_4_N_4_O_3_	10.845	0.271
29	1,4‐diaza‐2,5‐dioxobicyclo[4.3.0]nonane	C_7_H_14_N_2_	10.975	1.039
30	2‐linoleoylglycerol, 2TMS derivative	C_27_H_54_O_4_Si_2_	11.095	0.312
31	Cyclo(Pro‐Leu)	C_11_H_18_N_2_O_2_	11.297	0.453
32	Octadecanoic acid, ethyl ester	C_20_H_40_O_2_	11.566	1.343
33	1‐deoxy‐1‐nitroheptitol	C_7_H_15_NO_8_	11.881	5.866
34	Pentadecanoic acid, ethyl ester	C_17_H_34_O_2_	12.072	2.680
35	D‐glucoheptose	C_7_H_14_O_7_	12.078	13.582
36	Hexitol	C_6_H_14_O_6_	12.292	33.082
37	D‐mannose	C_6_H_12_O_6_	12.599	1.275
38	(Z,Z)‐6,9‐pentadecadien‐1‐ol	C_15_H_28_O	12.865	0.905
39	N‐(3‐methyl‐1,2,4‐oxadiazol‐5‐yl)‐1‐pyrrolidinecarboximidamide	C_8_H_13_N_5_O	12.994	0.285
40	Linolic acid	C_18_H_32_O_2_	13.066	10.374
41	Hexadecanoic acid, ethyl ester	C_18_H_36_O_2_	13.225	2.302
42	N‐[4‐(3‐hydroxy‐1‐pyrrolidinyl)‐2‐butynyl]‐n‐methylacetamide	C_11_H_18_N_2_O_2_	13.381	0.169
	**Total identified**			**91.576**

^a^ Molecular formula.

^b^ Retention time (minute).

^c^ Relative area percentage (peak area relative to the total peak area, %).

*Nigrospora* genus fungi are oleaginous and known for producing significant amounts of fatty compounds (Tonato et al., 2018; Tonato et al., 2019). This study found that mycelium contained both saturated and unsaturated fatty acids and their esters (21.68%), which are key to herbicidal activity. Studies have demonstrated phytotoxicity of fatty acid esters such as dodecanoic acid (Angel et al., 2019), oleic acid, and linoleic acid (Perumalsamy et al., 2015). In addition, pelargonic acid acts as a contact herbicide by disrupting membrane functions and causing desiccation by interrupting the water continuum in intercellular spaces (Campos et al., 2022). In general, fatty compounds have high solubility in the substances present on leaf surfaces, which contributes to their comparatively high phytotoxicity and allows weed control to be achieved with lower quantities of herbicide (Breeze, 1993; Chotsaeng et al., 2019).

### 3.6 Effect on photosynthesis pigment content and membrane integrity

**FIGURE 5:**
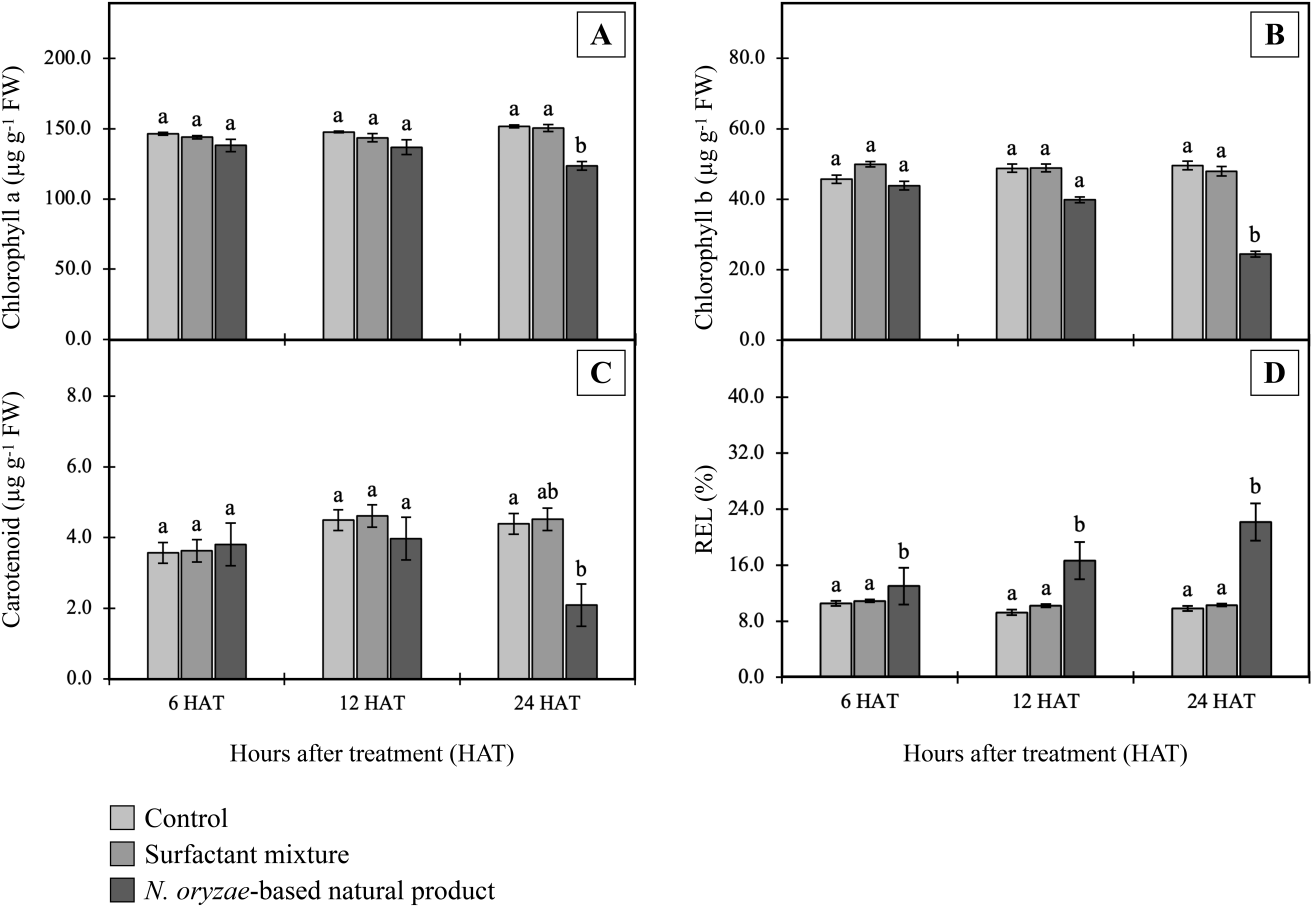
(A‐C) Photosynthesis pigment content and (D) relative electrolyte leakage (REL) of *E. crassipes* leaves treated with *N. oryzae*‐based natural product (8.0% w/v active ingredient). Means (n = 4) with different letters within a grouping are significantly different as indicated by Tukey's test (*p* < 0.05).

Treatment with 8.0% w/v *N. oryzae*‐based natural product induced clearly visible leaf burn symptoms in *E. crassipes* leaves even at 1 DAT (Figure 3A). These externally visible symptoms correspond with measurable physiological changes. Initially, chlorophyll *a*, chlorophyll *b*, and carotenoid contents showed no alteration in exposed leaves when compared to control plants (Figure 5A‐C). However, at 24 HAT, pigment levels decreased (*p*<0.05), indicating inhibition of photosynthesis due to pigment loss. Changes in the electrical conductivity values of treated plants are illustrated in Figure 5D. Electrolyte leakage increased significantly, indicating that the natural product disrupts membrane integrity, resulting in increased permeability and enhanced solute leakage. A *Citronella*‐based nano‐natural herbicide has similarly been shown to induce lipid peroxidation (Somala et al., 2023), which affects membrane bilayer phospholipids. In that study, loss of membrane integrity was determined to impact cell components (chlorophyll *a*, *b*, and carotenoid) and metabolic processes, ultimately resulting in slowed plant development and finally death within 21 days of treatment.

### 3.7 Effect on lipid peroxidation and ROS accumulation

**FIGURE 6:**
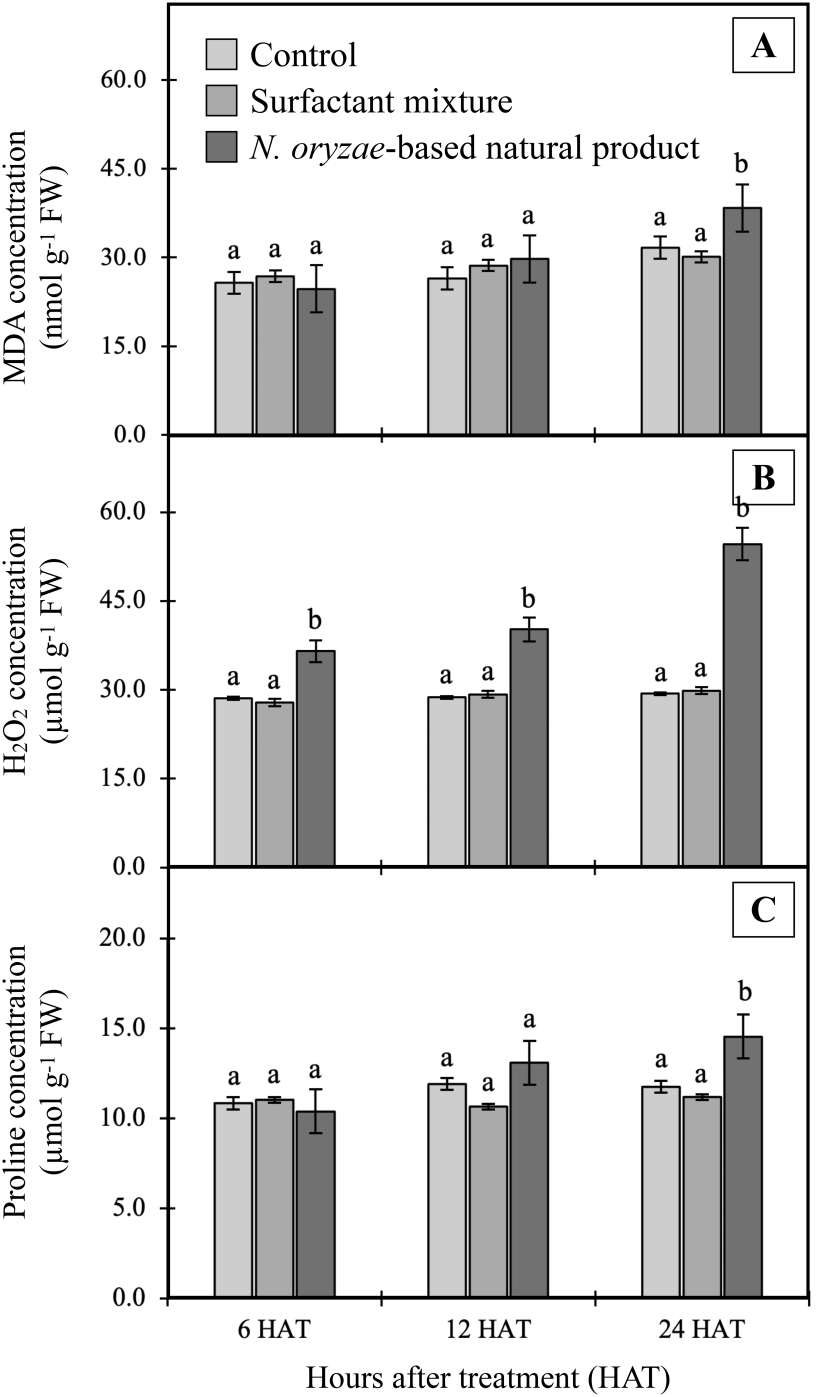
Effects of the *N. oryzae*‐based natural product (8.0% w/v active ingredient) on (A) H_2_O_2_, (B) MDA, and (C) proline concentration in *E. crassipes* leaves. Different letters within a time group indicate significant differences among treatment means based on Tukey's significant difference test (n = 4; *p* < 0.05).

Levels of H_2_O_2_, MDA, and proline in *E. crassipes* plants increased significantly (*p*<0.05) when exposed to 8% w/v concentrations of *N. oryzae*‐based natural product, with maximum respective increases of 46.26%, 17.49%, and 19.16% over control at 24 hours after treatment. The enhanced lipid peroxidation (Figure 6A) and increased H_2_O_2_ content (Figure 6B) in treated leaves indicate an increased generation of ROS in response to oxidative stress. Of the various ROS, H_2_O_2_ is abundant as a by‐product of aerobic metabolism (Zámocký et al., 2012). In plants, ROS levels can increase under both biotic and abiotic stresses, such as herbicide treatment (Traxler et al., 2023). Being a small, diffusible molecule that can readily cross biological membranes, H_2_O_2_ is of significant utility as an oxidative stress marker following herbicide application (Gaafar et al., 2022; Shopova et al., 2021; Agostinetto et al., 2016). Furthermore, although H_2_O_2_ can act as a beneficial signaling molecule in cellular cascades, excessive H_2_O_2_ content induces oxidative damage, leading to programmed cell death. This effect has been observed in numerous plants, including tobacco (Houot et al., 2001), *Arabidopsis praecox* (Zhang et al., 2015), and *Dendrobium nobile* (Hsu et al., 2022).

The increase in MDA levels indicates membrane damage due to peroxidation of polyunsaturated fatty acids (Montillet et al., 2005). Moreover, elevated REL (Figure 5D) thus also supports the induction of membrane damage by treatment with the *N. oryzae*‐based natural product; REL is an indicator of membrane damage due to peroxidation resulting from an oxidative burst (Singh et al., 2007).

Proline acts as a free radical scavenger, activates ROS detoxification pathways, and balances cell redox (Zulfiqar and Ashraf, 2023; María et al., 2021). In general, proline levels increase in glyphosate‐treated plants (Soares et al., 2019; Spormann et al., 2019), and the present results are consistent with those observations, with accumulation of proline (Figure 6C), suggesting a stress response in *E. crassipes* plants under *N. oryzae*‐based natural product treatment. The extract contains a diverse range of compounds, as shown in Table 3. Literature suggests that some of these compounds may influence oxidative parameters. GC‐MS data identifies the presence of fatty acids and their derivatives, which are known to induce oxidative stress by disrupting membrane stability and activating oxidative stress pathways (Cao et al., 2024). Pyrrolidin‐2‐one (Nosalova et al., 2025), L‐lysine (Seminotti et al., 2008), 2(3H)‐furanone, dihydro‐4‐hydroxy‐ (Chen et al., 2021), and lithic acid (Sautin et al., 2007) have also been reported to stimulate ROS production, thereby amplifying oxidative damage. This leads us to hypothesize that these constituents may contribute to the effects observed in this study.

### 3.8 Effect on antioxidant activity

**FIGURE 7:**
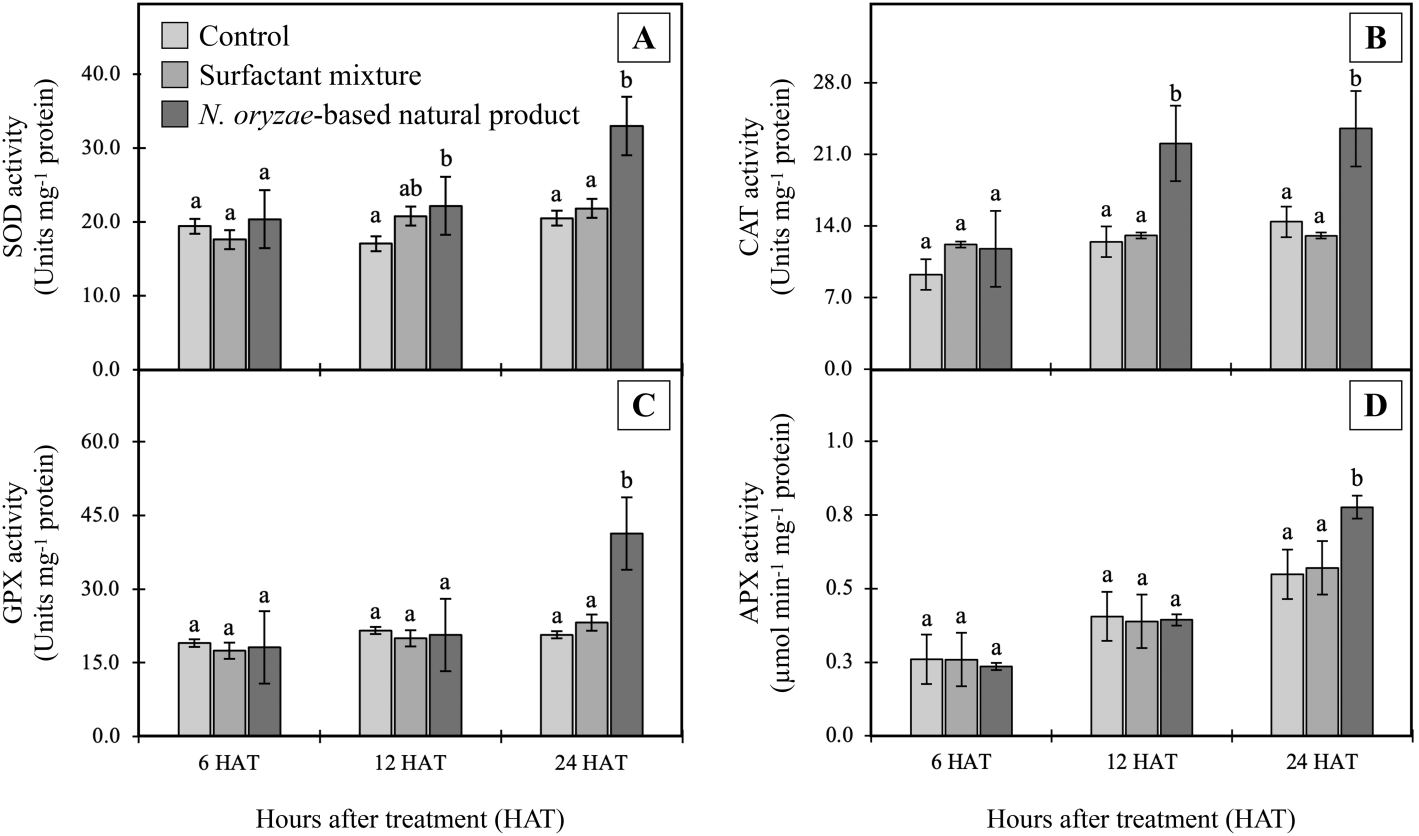
Effects of *N. oryzae*‐based natural product (8.0% w/v active ingredient) on (A) SOD, (B) CAT, (C) GPX, and (D) APX activity of *E. crassipes* leaves. Different letters within a time group indicate significant differences among treatment means based on Tukey's significant difference test (n = 4; *p* < 0.05).

Plant cells require a homeostatically controlled redox potential for maintenance of normal metabolism, which is ensured by antioxidant defense systems consisting of ROS‐removing molecules and various redox enzymes (Breitenbach et al., 2015). In this study, antioxidant enzyme activities were found to be significantly increased in *E. crassipes* leaves treated with the *N. oryzae*‐based natural product (8.0% w/v active ingredient) (Figure 7). All enzyme activities followed similar upward trends, with peak activities at 24 HAT. The greatest increase was observed for SOD, which rose 46.15% over the control group (*p*<0.05); meanwhile, APX, GPX, and CAT exhibited increases of 42.86%, 38.89%, and 35.71%, respectively. These relatively consistent increases in antioxidant enzyme activities, along with enhanced stress marker levels as described above, provide strong evidence that oxidative stress occurs in treated *E. crassipes*. Previous research has shown enhanced antioxidant enzyme activity in plants treated with glyphosate (Sharma et al., 2016), paraquat, atrazine, and 2,4‐D (Kaur, 2019). Upregulation of these enzymes implies that the plant is attempting to eliminate high ROS concentrations and protect against damage caused by oxidative processes (Shopova et al., 2021). However, the observed membrane damage (Figure 5D) and increased lipid peroxidation (Figure 6A) suggest that despite this upregulation, the plant's defense mechanisms remain overwhelmed. Oxidative stress likely plays a major role in disrupting cell membrane integrity, leading to the observed cellular damage (Breitenbach et al., 2015). The resulting membrane dysfunction can severely impair metabolic processes, including photosynthesis, as evidenced by the reduced chlorophyll content. Loss of chlorophyll can be used for monitoring damage to plant growth and development, which results in tissue discoloration, necrosis, desiccation, or death (Kim et al., 2004; Song et al., 2007). All together, these findings demonstrate that oxidative stress may be a key mechanism through which the *N. oryzae*‐based natural product exerts its herbicidal effects on *E. crassipes*. Although the individual effects of the compounds on oxidative and antioxidant parameters remain unclear based on the current study, the literature indicates that the identified compounds are known to influence ROS production. These effects may also indirectly modulate antioxidant enzyme activities. It is plausible that the observed effects result from the joint action of multiple components rather than the activity of a single dominant compound, as is often the case with natural crude extracts (Bordin et al., 2021; Cimmino et al., 2015). Further investigations using purified fractions or individual compounds from the extract would be valuable to elucidate their specific contributions and interactions in inducing oxidative damage or modulating antioxidant parameters.

## 4 CONCLUSION

This research demonstrates the strong potential of *N. oryzae* crude extract as an active ingredient for the development of a natural herbicide against *E. crassipes* macrophytes. The extract showed effective control activity and multiple modes of action that may decrease herbicide resistance development. All three modes, namely cellular damage, photosynthesis disruption, and oxidative stress induction, are indicators of phytotoxicity. Future studies should concentrate on experiments to assess efficacy in field conditions and the impact on non‐target organisms in lentic ecosystems. Nonetheless, in light of the growing global trend towards restricting chemical herbicide use, this fungus‐based approach for deriving herbicidal products presents a promising alternative with a potentially eco‐friendly active ingredient source and a sustainable strategy for future aquatic weed management.

## AUTHOR CONTRIBUTIONS

**CL:** Conceptualization, Funding acquisition, Resources, Supervision, Writing‐review and editing. **NM:** Conceptualization, Investigation, Project administration, Writing‐original draft. **PW:** Investigation, Methodology, Formal analysis, Writing‐original draft. **KY:** Formal analysis, Validation, Writing‐review and editing. **MT:** Formal analysis, Validation. **HP:** Formal analysis, Validation.

## FUNDING

National Science, Research and Innovation Fund (NSRF; Grant number RE‐KRIS/FF67/026), Thailand

## CONFLICT OF INTEREST STATEMENT

The authors declare no conflict of interest

## Data Availability

DNA sequence data of *Nigrospora oryzae* 016 from this research have been deposited in the NCBI repository (https://www.ncbi.nlm.nih.gov/) with the following accession numbers: *ITS* (PP532837), *TEF‐1* (PP565361), and *TUB2* (PP565362).
